# Comparison of Protein *N*-Homocysteinylation in Rat Plasma under Elevated Homocysteine Using a Specific Chemical Labeling Method

**DOI:** 10.3390/molecules21091195

**Published:** 2016-09-08

**Authors:** Tianzhu Zang, Ligi Paul Pottenplackel, Diane E. Handy, Joseph Loscalzo, Shujia Dai, Richard C. Deth, Zhaohui Sunny Zhou, Jisheng Ma

**Affiliations:** 1 Barnett Institute of Chemical and Biological Analysis, Department of Chemistry and Chemical Biology, Northeastern University, Boston, MA 02115, USA; zangt@mail.med.upenn.edu (T.Z.); daishujia@gmail.com (S.D.); 2 Human Nutrition Research Center on Aging, Tufts University, Boston, MA 02111, USA; Ligi.Paul_Pottenplackel@tufts.edu; 3 Department of Medicine, Brigham and Women’s Hospital, Harvard Medical School, Boston, MA 02115, USA; dhandy@rics.bwh.harvard.edu (D.E.H.); jloscalzo@partners.org (J.L.); 4 Department of Pharmaceutical Sciences, Nova Southeastern University, Fort Lauderdale, FL 33314, USA; rdeth@nova.edu; 5 School of Pharmaceutical Sciences, Wenzhou Medical University, Wenzhou 325035, China

**Keywords:** hyperhomocysteinemia, cardiovascular disease, neuropsychiatric disease, protein *N*-homocysteinylation, plasma, biotin-aldehyde, Western blotting

## Abstract

Elevated blood concentrations of homocysteine have been well established as a risk factor for cardiovascular diseases and neuropsychiatric diseases, yet the etiologic relationship of homocysteine to these disorders remains poorly understood. Protein *N*-homocysteinylation has been hypothesized as a contributing factor; however, it has not been examined globally owing to the lack of suitable detection methods. We recently developed a selective chemical method to label *N*-homocysteinylated proteins with a biotin-aldehyde tag followed by Western blotting analysis, which was further optimized in this study. We then investigated the variation of protein *N*-homocysteinylation in plasma from rats on a vitamin B_12_ deficient diet. Elevated “total homocysteine” concentrations were determined in rats with a vitamin B_12_ deficient diet. Correspondingly, overall levels of plasma protein *N*-homocysteinylation displayed an increased trend, and furthermore, more pronounced and statistically significant changes (e.g., 1.8-fold, *p*-value: 0.03) were observed for some individual protein bands. Our results suggest that, as expected, a general metabolic correlation exists between “total homocysteine” and *N*-homocysteinylation, although other factors are involved in homocysteine/homocysteine thiolactone metabolism, such as the transsulfuration of homocysteine by cystathionine β-synthase or the hydrolysis of homocysteine thiolactone by paraoxonase 1 (PON1), may play more significant or direct roles in determining the level of *N*-homocysteinylation.

## 1. Introduction

**Figure 1:**
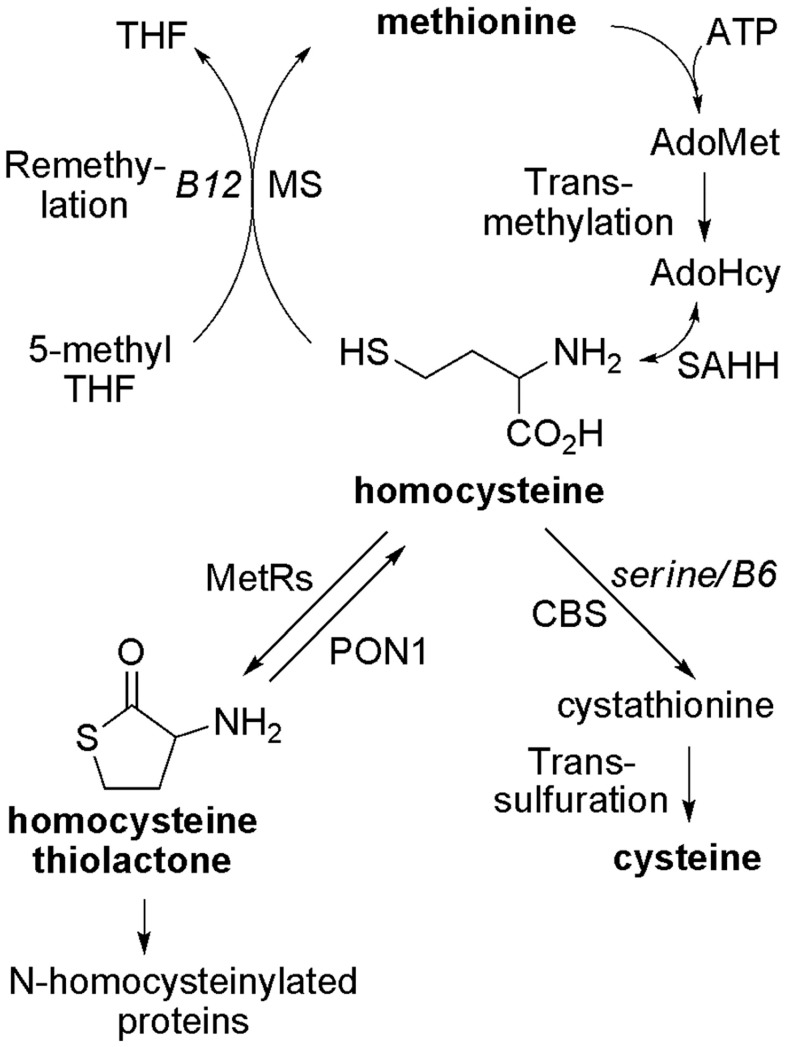
Metabolism of homocysteine in mammals. AdoHcy, *S*-adenosylhomocysteine; AdoMet, *S*-adenosylmethionine; ATP, adenosine-5′-triphosphate; CBS, cystathionine β-synthase; Hcy, homocysteine; Hcy TL, homocysteine thiolactone; MetRS, methionyl-tRNA synthetase; MS, methionine synthase; PON1, paraoxonase 1; SAHH, *S*-adenosylhomocysteine hydrolase; THF, tetrahydrofolate.

An abnormally increased concentration of homocysteine (Hcy) in blood or urine, i.e., hyperhomocysteinemia or homocystinuria, has been well recognized as a risk factor for cardiovascular, neuropsychiatric diseases, and other conditions [1,2,3,4,5,6]. As illustrated in Figure 1, in mammals, Hcy can be metabolized through two main pathways: methylation and transsulfuration [7,8,9,10,11]. In addition, Hcy can also be converted back to *S*-adenosyl-homocysteine (AdoHcy or SAH) because of the reversible transformation catalyzed by AdoHcy hydrolase (EC 3.3.1.1) [12,13]. It has been reported that methionine-rich diets (e.g., animal proteins), genetic defects of enzymes such as cystathionine β-synthase (CBS), deficiencies of nutritional factors (folate, vitamin B_6_ and B_12_), or a combination of such factors can lead to an increase of plasma “total homocysteine” (“total Hcy”) [7,8,14,15,16,17,18,19], which includes protein *S*-homocysteinylation (disulfides) and some free small molecule forms (e.g., free Hcy and its mixed disulfides with cysteine or glutathione) [3,20]. However, the pathophysiological consequences of homocysteinemia remain unclear.

**Figure 2:**
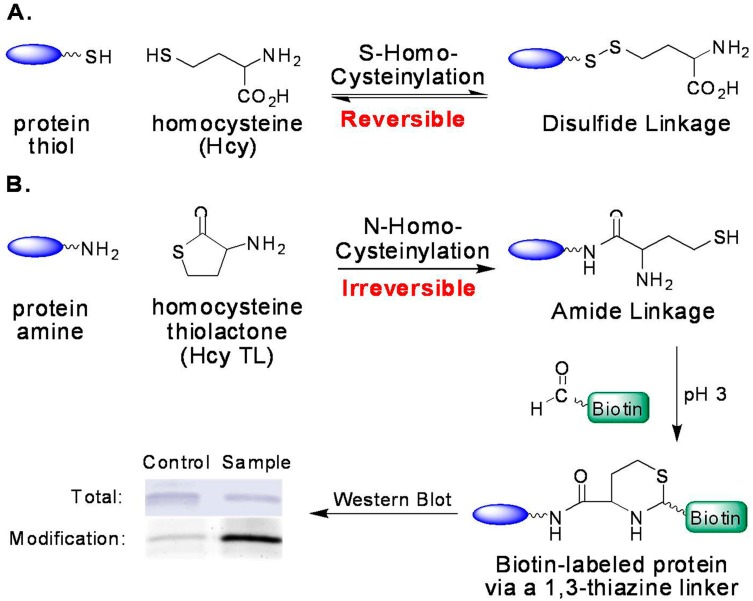
Formation of protein homocysteinylation and detection of *N*-homocysteinylation via selective tagging with aldehydes. (**A**) Reversible formation of *S*-homocysteinylation (disulfide); and (**B**) irreversible formation of *N*-homocysteinylation (amide) and its detection.

Possible mechanisms of Hcy toxicity have been proposed, such as the induction of oxidative stress and the ensuing-alteration of protein structure and loss of protein function, or the inhibition of transmethylation by the accumulation of AdoHcy—the common methylation product and a potent inhibitor for most methyltransferases [21,22,23,24,25,26]. Another possibility is that homocysteine is covalently attached to proteins, including *S*-homocysteinylation by forming mixed disulfides with cysteine residues and *N*-homocysteinylation by forming homocystamides (Figure 2) [27,28,29]. Protein *S*-homocysteinylation has been identified in several plasma proteins, for example, *S*-homocysteinylated transthyretin [30,31]. It is worth noting that “total Hcy” is a misnomer (historical usage), as it includes more than 70% of protein *S*-homocysteinylation (disulfides), but excludes other important Hcy species, such as protein *N*-homocysteinylation in human plasma [3,20,22]. First reported by Jakubowski, protein *N*-homocysteinylation results from the non-enzymatic acylation of the amino groups in proteins (either on the side chain of lysines and/or the N-termini) by homocysteine thiolactone (Hcy thiolactone or Hcy TL), which is produced as a byproduct from the editing process of methionyl-tRNA synthetase (Figure 1 and Figure 2) [29,32,33,34].

In comparison to *S*-homocysteinylation, protein *N*-homocysteinylation is irreversible (chemically stable with no enzyme found to reverse the process) and, thus, may accumulate in proteins, particularly in long-lived ones [35,36]. In human plasma, *N*-homocysteinylation was reported ~0.2–0.5 μM, and the ratio of “total Hcy” to total *N*-homocysteinylation was found to be 7:1 to 10:1 [27,37,38]. Several human proteins have been examined more closely; human serum albumin and hemoglobin contain 0.3% and 0.6% *N*-homocysteinylation (mol of modification/mol of total protein), and 0.06% and 1.0% *S*-homocysteinylation, respectively [39]. Another publication reported that the levels of *N*-homocysteinylated (*N*-Hcy) proteins in plasma were increased due to the mutations or deletions in the cystathionine β-synthase (CBS) or methylenetetrahydrofolate reductase genes in mice [40].

Like other protein modifications, protein *N*-homocysteinylation may affect protein cleavage, crosslinking, aggregation, autoimmune response, and function [41,42,43,44,45,46,47,48,49,50,51,52]. In addition, thiols are highly reactive and may undergo myriad transformations under physiological conditions (such as redox, alkylation, and even desulfurization) [53,54,55,56,57]. One example is homocystamide-induced protein oxidative damage by triggering the formation of free radicals, discovered by Strongin’s laboratory [58]. It has also been observed that the formation of protein aggregates from *N*-homocysteinylated acidic proteins (e.g., α-lactalbumin) induces the tertiary structural changes and functional alterations [59]. Recently, it was reported that *N*-Hcy proteins affected gene expression in human vascular endothelial cells, which is related to cardiovascular development and neurological disease [60]. Thus, Jakubowski and others have hypothesized that protein *N*-homocysteinylation is an important contributor to the pathological consequences of hyperhomocysteinemia, either together with or independent of elevated concentrations of plasma “total Hcy” (free Hcy and disulfides) [29,39,45,61].

Methods for the quantification of *N*-Hcy proteins have been developed. The first and most commonly used is the complete chemical hydrolysis of proteins, followed by subsequent analysis of free homocysteine using HPLC coupled with fluorescence or UV detection [37,62]; however, the modified sites cannot be identified based on current methods of amino acid analysis. Alternatively, an immunological assay (ELISA and dot blotting) using polyclonal antibodies was reported [36,63], but the specificity remains to be established.

Until now, lack of a systematic analysis of *N*-Hcy proteins in complex systems has prevented a more thorough understanding of molecular and pathobiological consequences in hyperhomocysteinemia. Toward this end, we have developed a chemical method to selectively derivatize *N*-Hcy groups with different aldehyde tags under mildly acidic conditions [64]. For example, by introducing a biotin-containing aldehyde tag onto the *N*-homocystamide group (Figure 2B), Western blotting coupled with a chemiluminescence assay can be used to both detect and quantify *N*-homocysteinylation of different proteins. In comparison to antibodies against *N*-Hcy proteins, the biotin-aldehyde is commercially available and inexpensive. Hence, our method makes it feasible for global profiling and quantitative analysis of *N*-Hcy proteins in complex systems [64], including proteomic studies from other laboratories [65,66]. In this study, we investigated changes in protein *N*-homocysteinylation associated with variation of “total Hcy” concentrations in plasma from rats. Taken together, this proteomic study reveals that protein *N*-homocysteinylation may be affected by homocysteine metabolism and, furthermore, opens new avenues by which to discover potential biomarkers and to understand better the underlying molecular mechanisms.

## 2. Results and Discussions

### 2.1. Optimized Conditions for Aldehyde Tag Labeling

**Figure 3:**
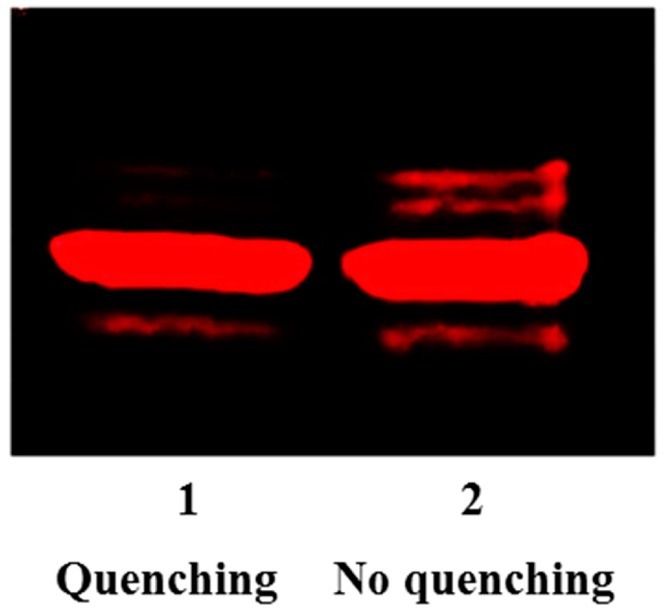
Fluorescence intensities of rhodamine-aldehyde labeled *N*-Hcy myoglobin with and without cysteamine quenching reaction. Lane 1: 7.8 µM modified myoglobin including 3.1 µM *N*-Hcy myoglobin and 4.7 µM native myoglobin with cysteamine (500 µM) quenching; Lane 2: 7.8 µM modified myoglobin including 3.1 µM *N*-Hcy myoglobin and 4.7 µM native myoglobin without cysteamine quenching.

As we previously demonstrated, the coupling between aldehydes and *N*-homocystamide is highly specific under mildly acidic conditions (pH 2 to 4), because competing amines are protonated and rendered inactive [64,67]. However, the pH values of the loading buffer and running buffer for sodium dodecyl sulfate polyacrylamide gel electrophoresis (SDS-PAGE) are around 6.8 and 8.3, respectively; under these conditions, significant percentages of amines in proteins are present in the neutral form, favoring the formation of a protein Schiff base with the excess aldehyde labeling reagent. Hence, in this study, cysteamine was added to the solution to quench the excess aldehyde reagent. As a beta-amino thiol, cysteamine reacts with aldehyde to form 1,3-thiazolidine at pH 3.0 (Figure S2 in Supplementary Materials). Under our conditions, after quenching with cysteamine, no aldehyde reagent was left in the solution to react with free amino groups during the subsequent sample handling steps, thereby eliminating non-specific labeling (Figure 3). Moreover, compared to antibody-based assays, our labeling method uses a commercially available biotin-aldehyde and streptavidin without the need of more expensive primary and secondary antibodies, and moreover, only requires one-step Western blotting (i.e., no need for secondary antibodies). Altogether, our method significantly reduces both cost and time, and also improves both accuracy and reproducibility.

### 2.2. Increased Level of Protein N-Homocysteinylation in Plasma from Rats on B_12_ Deficient Diet

**Figure 4:**
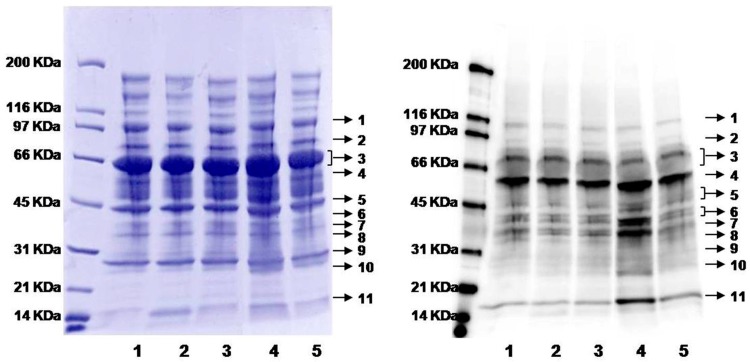
Gel images of plasma proteins from rats on B_12_-deficient and control diets. Left image: Coomassie blue staining showing total protein loading; right image: chemiluminescence from Western blotting showing the levels of biotin-labeling (protein *N*-homocysteinylation). Lanes 1, 2, and 3: plasma from individual rats on control diet; lanes 4 and 5: plasma from individual rats on a B_12_-deficient diet. Proteins were divided into eleven individual protein bands (indicated by the arrows) for subsequent analysis.

As described in Figure 1, nutrients such as folate, vitamin B_12_ and B_6_ affect the concentration of homocysteine [7,17]. A diet deficient in vitamin B_12_ has been observed to cause hyperhomocysteinemia in humans and animals [68,69,70,71]. Rats on vitamin B_12_-deficient or control diets (six each) were analyzed in this work. The plasma concentration of “total Hcy” was 31.1 ± 10.0 (standard deviation, SD) and 4.7 ± 0.8 µM for rats on B_12_-deficient and control diets, respectively (Table 1), a difference of 6.6-fold. As expected, the overall level of protein *N*-homocysteinylation shows an increased trend with 1.3-fold rising (see overall intensity in Table 1 and Figure S3), while the abundance of plasma proteins (total and individual) remained about the same (Coomassie blue image in Figure 4). More strikingly, several individual bands displayed much larger differences in protein modifications (Figure 4 and Table 1). For example, normalized to the control group (1.0 ± 0.4), band 11 has a ratio of 1.8 ± 0.7 for B_12_-defecient rats with a *p*-value of 0.03 (Table 1). Moreover, it should be pointed out that plasma samples were blinded to treatment and the nature of the samples revealed only after all analysis had been completed as shown above. Thus, our method is well suited for large scale quantification of protein *N*-homocysteinylation in biological samples. So far, this method has been applied to determine the relative levels of *N*-homocysteinylation in serum from autistic children with elevated levels of Hcy to investigate the relationship between *N*-homocysteinylation and neurophysiological disorders [66], in embryos from mice with the folate-responsive neural tube defects to check the correlation between folate and *N*-homocysteinylation in the embryos [65], and in plasma from mice with heterozygous deficiency (*Cbs*^+/−^) of cystathionine β-synthase (CBS) to study the correlation between CBS deficiency and protein *N*-homocysteinylation (preliminary data from our laboratories shown in Figure S4) [72,73]. Further studies would be focused on the identification of *N*-Hcy proteins and modified sites in order to discover the potential *N*-Hcy protein biomarkers. This assay could be accomplished by enrichment of *N*-Hcy protein digests using aldehyde resin coupled with liquid chromatography tandem mass spectrometry (LC-MS/MS) analysis [64]. For future work, quantitative mass spectrometry approaches such as isotopic labeling (e.g., iTRAQ) can be combined with ours [74]. Such a combination should also allow us to perform both identification of the modified sites and quantification at the same time.

### 2.3. Possible Contributing Factors to N-Homocysteinylation

As shown in our work, whereas there were increases in *N*-homocysteinylation when the blood concentrations of “total Hcy” (disulfides) were elevated, the magnitude of the former is smaller. This finding is not unexpected, considering the formation of “total Hcy” (free Hcy and disulfides) and *N*-homocysteinylation are influenced by multiple and different factors. As illustrated in Figure 1, except the nutritional factors, the activities of different enzymes which involve in the conversion of Hcy to methionine, cysteine and Hcy thiolactone could also contribute to the variation of protein *N*-homocysteinylation. Hcy thiolactone can be hydrolyzed back to Hcy by paraoxonase 1 (PON1, EC 3.1.8.1) [35]. Therefore, activity of PON1 likely plays a direct and significant role in Hcy thiolactone metabolism and, hence, *N*-homocysteinylation. For example, PON1 is present in serum, and there is little protein synthesis in serum (hence, little formation of Hcy thiolactone) [75,76]. Thus, the steady-state concentration of Hcy thiolactone is extremely low (0.1–26 nM), while “total Hcy” is in the 5–15 µM range [22]; as such, the level of *N*-homocysteinylation is expected to be lower than the “total Hcy”, as we observed here. Conversely, in tissues and within cellular compartments, where protein synthesis is more active, Hcy thiolactone formation is likely to be higher as well. Ultimately, the counterbalance of the formation and hydrolysis of Hcy thiolactone, not simply the concentration of homocysteine, determines the level of *N*-homocysteinylation. In addition, as mentioned in Section 1, the inactivation of cystathionine β-synthase (CBS) in transgenic mice (*Cbs^−/−^*) caused 50- to 140-fold elevation of “total Hcy” and six- to 10-fold increase of *N*-Hcy proteins in serum [40]. As such, patients with genetically-deficient enzymes could cause a more striking increase of *N*-Hcy proteins, and *N*-Hcy proteins would be more effectively identified using LC-MS/MS. Recently, *N*-Hcy fibrinogen and its modified sites have been identified in the plasma from CBS-deficient patients [77]. Based on our results and the published reports, we propose that differences on enzyme activity (e.g., PON1 and CBS) are likely to alter plasma *N*-homocysteinylation status more distinctly, which can be tested using our method. Finally, given the simplicity, low-cost, and robustness of our method, our method can be easily employed for clinical applications [66].

## 3. Materials and Methods

### 3.1. Plasma

Plasma from rats on vitamin B_12_-deficient and control diets (six in each group) were from Dr. Ligi Paul at Tufts University. All animal procedures were approved by the Institutional Animal Care and Use Committee of the Jean Mayer USDA Human Nutrition Research Center on Aging at Tufts University (Protocol No. SE-56) and conducted according to the Guide for the Care and Use of Laboratory Animals (1996). Measurement of “total Hcy” in plasma was performed as reported [78]. Protein concentrations of plasma were assayed according to the Bradford method using concentrated dye (500-0006) from Bio-Rad (Hercules, CA, USA); and bovine serum albumin (A3059) purchased from Sigma-Aldrich (St. Louis, MO, USA) was used as a standard.

### 3.2. Chemicals and Reagents

Horseradish peroxidase streptavidin (SA-5004) was from Vector Laboratories (Burlingame, CA, USA). SuperSignal West Pico chemiluminescent substrate was from Thermo Scientific (Rockford, IL, USA). Myoglobin from equine skeletal muscle (M0630) was from Sigma-Aldrich. *N*-Homocysteinylated myoglobin was prepared as we previously reported [64]. Biotinyl-Asp-Glu-Val-Asp-aldehyde (CAS registry number: 178603-73-1) was from Bachem Americas (N-1470, Torrance, CA, USA; see Figure S1 for its structure in Support Information). Rhodamine-aldehyde (10 mM) in 50% 200-proof ethanol was synthesized as previously described [64]. EZ-Run prestained protein ladder (BP3603) was from Fisher Scientific. Natural unstained protein ladder (161-0317) was from Bio-Rad. Biotinylated protein markers were from Sigma-Aldrich (B2787) and Bio-Rad (161-0319), respectively. All reagents were ACS grade and used as received without further purification. All incubations were carried out in an Eppendorf Thermomixer^®^ (Eppendorf North America, Hauppauge, NY, USA) at 25 °C unless specified otherwise.

### 3.3. Optimization of Labeling with Cysteamine Quenching

Hcy thiolactone-modified myoglobin (13 µM including 5.2 µM *N*-Hcy myoglobin (~40%) and 7.8 µM native myoglobin) was incubated with 200 µM Rhodamine-aldehyde (pH 3), containing 50 mM citric acid, 500 µM tris(2-carboxyethyl)phosphine (TCEP), at 25 °C in the dark for 8 h [64]. Aliquots (20 µL) were removed and stored at 4 °C. Cysteamine (15 mM, 1 µL) was added to the remaining solution to quench the excess aldehyde for 3–14 h. Solutions (12 µL) with or without cysteamine quenching were mixed with loading buffer (8 µL, 5% SDS, and 25% glycerol) for SDS-PAGE analysis. Gels were analyzed using a Molecular Dynamics Storm840 imaging system (GE Healthcare, Piscataway, NJ, USA). Fluorescence was recorded with an excitation wavelength at 450 nm and an emission wavelength at 520 nm using a Strom Scanner Control version 5.03 (Amersham Bioscience, Piscataway, NJ, USA), and data were visualized by ImageQuant TL 7.0 (GE Healthcare, Pittsburgh, PA, USA).

### 3.4. Labeling Proteins with Biotin-Aldehyde

Rat plasma (2 mg/mL protein from each sample, final concentration) was incubated with 250 µM biotin-aldehyde in 200 mM citric acid, 2 mM TCEP, pH 3, in the dark at 25 °C for 5 h. To quench the labeling reaction, cysteamine (1 mM, final concentration) in 50 mM citric acid and 2 mM TCEP, pH 3, was added to each sample and incubated for additional 3 h. Reaction solutions were stored at −80 °C before analysis.

### 3.5. Western Blotting of Biotin-Labeled Proteins

Labeling reactions (35 μL) were mixed with 2× Laemmli loading buffer (35 µL) containing 350 mM dithiothreitol (DTT) and then boiled in water for 5 min. For each reaction, two aliquots (30 µL) were loaded into two separate precast Tris-HCl gels (4%–15%, Bio-Rad) for sodium dodecyl sulfate polyacrylamide gel electrophoresis (SDS-PAGE). One gel was used for Coomassie blue staining and the other for Western blotting. For blotting, proteins from the gel were transferred onto an Immun-Blot PVDF Membrane (0.2 µm, Bio-Rad) using transfer buffer (25 mM Tris, 192 mM glycine, 20% methanol, pH 8.3). The membranes were next blocked in 2% bovine serum albumin (BSA) in TBST (25 mM Tris, 137 mM NaCl, 3 mM KCl, 0.1% Tween-20, pH 7.4) for 1 h. After blocking, the membrane was washed with TBST for 3 × 10 min and incubated with 0.5 µg/mL streptavidin-horseradish peroxidase (HRP) in 20 mL TBST for 1 h. The membrane was then washed again in TBST for 5 × 6 min and incubated in PBS (68 mM NaCl, 1 mM KCl, 5 mM Na_2_HPO_4_, and 1 mM KH_2_PO_4_, pH 7.4) for 10 min. After incubation, the buffer was discarded, and the chemiluminescence signal was developed by the addition of 1 mL SuperSignal West Pico chemiluminescent substrate for 1 min. Chemiluminescence was detected by FluorChem Imager SP (Alpha Innotech, San Leandro, CA, USA), and the image was analyzed by ImageQuant TL 7.0 (GE Healthcare). The experiment was conducted in duplicate for each sample.

### 3.6. Data Analysis for the Degree of Modification

Protein *N*-homocysteinylation in overall level and in each protein band's level (See Figure 4) were determined from the chemiluminescent intensity, which was normalized by using the Coomassie blue staining intensity of overall protein bands in order to minimize the intensity variation from the protein loading amount. Eventually, the normalized chemiluminescent intensity was compared to the control group and the statistical analysis was performed using two-tailed *t*-test analysis by GraphPad Prism 6 (GraphPad Software Inc., La Jolla, CA, USA).

## 4. Conclusions

For the first time, global analysis of protein *N*-homocysteinylation was performed in the plasma from rats with perturbed homocysteine metabolism. As expected, a general correlation between “total homocysteine” and *N*-homocysteinylation was observed. Interestingly, more pronounced and statistically significant changes were identified for some individual protein bands. While larger sets of samples should be analyzed before a conclusive interpretation can be drawn, our method has been shown to be suitable for quantitative analysis. Moreover, our results suggest that other factors directly involved in homocysteine/homocysteine thiolactone metabolism, such as the activity of cystathionine β-synthase (CBS) or paraoxonase 1 (PON1), may play more direct and pronounced roles in *N*-homocysteinylation and facilitate the identification of *N*-Hcy proteins.

## Figures and Tables

**Table 1:** Chemiluminescence intensities of biotin-labeled proteins (*N*-homocysteinylation) and “total homocysteine” concentration of rat plasma.

	B12 Deficiency *	Control Diet	
	Chemiluminescence Intensity		
Band	Mean ± S.D.	Mean ± S.D.	*p*-Value
1	1.2 ± 0.3	1.0 ± 0.4	0.3
2	1.3 ± 0.4	1.0 ± 0.3	0.2
3	1.3 ± 0.4	1.0 ± 0.3	0.2
4	1.2 ± 0.3	1.0 ± 0.2	0.2
5	1.2 ± 0.2	1.0 ± 0.2	0.09
6	1.3 ± 0.2	1.0 ± 0.2	0.03
7	1.4 ± 0.3	1.0 ± 0.2	0.009
8	1.4 ± 0.2	1.0 ± 0.3	0.04
9	1.2 ± 0.3	1.0 ± 0.3	0.2
10	1.5 ± 0.6	1.0 ± 0.4	0.09
11	1.8 ± 0.7	1.0 ± 0.4	0.03
Overall	1.3 ± 0.3	1.0 ± 0.2	0.08
	“Total homocysteine” (µM)		
	31.1 ± 10.0	4.7 ± 0.8	5.00 × 10^−5^

*: six samples in each group were analyzed in duplicate; overall intensity is for all proteins in the complete lanes. Intensities have been normalized to those from control diet; *p*-value < 0.05 is considered a statistically significant change (highlighted in italic); S.D.: standard deviation.

## Supplementary Materials

Supplementary materials can be accessed at: http://www.mdpi.com/1420-3049/21/9/1195/s1.

## References

[1] Brattstrom L., Wilcken D.E. (2000). Homocysteine and cardiovascular disease: Cause or effect?. Am. J. Clin. Nutr..

[2] Cacciapuoti F. (2011). Hyper-homocysteinemia: A novel risk factor or a powerful marker for cardiovascular diseases? Pathogenetic and therapeutical uncertainties. J. Thromb. Thrombolysis.

[3] Carmel R.J., Jacobsen D.W. (2001). Homocysteine in Health and Disease.

[4] Maron B.A., Loscalzo J. (2009). The treatment of hyperhomocysteinemia. Annu. Rev. Med..

[5] Seshadri S., Beiser A., Selhub J., Jacques P.F., Rosenberg I.H., D’Agostino R.B., Wilson P.W., Wolf P.A. (2002). Plasma homocysteine as a risk factor for dementia and Alzheimer’s disease. N. Engl. J. Med..

[6] Diaz-Arrastia R. (2000). Homocysteine and neurologic disease. Arch. Neurol..

[7] Selhub J. (1999). Homocysteine metabolism. Annu. Rev. Nutr..

[8] Finkelstein J.D. (2000). Pathways and regulation of homocysteine metabolism in mammals. Semin. Thromb. Hemost..

[9] Matthews R.G., Smith A.E., Zhou Z.S., Taurog R.E., Bandarian V., Evans J.C., Ludwig M. (2003). Cobalamin-dependent and cobalamin-independent methionine synthases: Are there two solutions to the same chemical problem?. Helv. Chim. Acta.

[10] Gui S., Wooderchak-Donahue W.L., Zang T., Chen D., Daly M.P., Zhou Z.S., Hevel J.M. (2013). Substrate-induced control of product formation by protein arginine methyltransferase 1. Biochemistry.

[11] Zhou Z.S., Peariso K., Penner-Hahn J.E., Matthews R.G. (1999). Identification of the zinc ligands in cobalamin-independent methionine synthase (MetE) from *Escherichia coli*. Biochemistry.

[12] Mosley S.L., Bakke B.A., Sadler J.M., Sunkara N.K., Dorgan K.M., Zhou Z.S., Seley-Radtke K.L. (2006). Carbocyclic pyrimidine nucleosides as inhibitors of *S*-adenosylhomocysteine hydrolase. Bioorg. Med. Chem..

[13] Deth R.C., Hodgson N.W., Trivedi M.S., Muratore C.R., Waly M.I. (2011). Autsim: A Neuroepigenetic Disorder. Autsim Sci. Dig..

[14] Blom H.J.C., Fowler B., Koch H.G. (1998). Disorders of homocysteine metabolism: From rare genetic defects to common risk factors. Eur. J. Pediatr..

[15] Goyette P., Sumner J.S., Milos R., Duncan A.M., Rosenblatt D.S., Matthews R.G., Rozen R. (1994). Human methylenetetrahydrofolate reductase: Isolation of cDNA mapping and mutation identification. Nat. Genet..

[16] Mudd S.H., Finkelstein J.D., Irreverre F., Laster L. (1964). Homocysteinuria: An enzymatic defect. Science.

[17] Selhub J., Jacques P.F., Wilson P.W., Rush D., Rosenberg I.H. (1993). Vitamin status and intake as primary determinants of homocysteinemia in an elderly population. J. Am. Med. Assoc..

[18] Verhoef P., van Vliet T., Olthof M.R., Katan M.B. (2005). A high-protein diet increases postprandial but not fasting plasma total homocysteine concentrations: A dietary controlled, crossover trial in healthy volunteers. Am. J. Clin. Nutr..

[19] Handy D.E., Loscalzo J. (2003). Homocysteine and atherothrombosis: Diagnosis and treatment. Curr. Atheroscler. Rep..

[20] Jakubowski H. (2013). An overview of homocysteine metabolism. Homocysteine in Protein Structure/Function and Human Disease: Chemical Biology of Homocysteine-Containing Proteins.

[21] Olszewski A.J., McCully K.S. (1993). Homocysteine metabolism and the oxidative modification of proteins and lipids. Free Radic. Biol. Med..

[22] Perla-Kajan J., Twardowski T., Jakubowski H. (2007). Mechanisms of homocysteine toxicity in humans. Amino Acids.

[23] Perna A.F., Ingrosso D., Lombardi C., Acanfora F., Satta E., Cesare C.M., Violetti E., Romano M.M., De Santo N.G. (2003). Possible mechanisms of homocysteine toxicity. Kidney Int. Suppl..

[24] Cannon L.M., Butler F.N., Wan W., Zhou Z.S. (2002). A stereospecific colorimetric assay for (*S*,*S*)-adenosylmethionine quantification based on thiopurine methyltransferase-catalyzed thiol methylation. Anal. Biochem..

[25] Biastoff S., Teuber M., Zhou Z.S., Drager B. (2006). Colorimetric activity measurement of a recombinant putrescine N-methyltransferase from Datura stramonium. Planta Med..

[26] Joseph J., Joseph L., Devi S., Kennedy R.H. (2008). Effect of anti-oxidant treatment on hyperhomocysteinemia-induced myocardial fibrosis and diastolic dysfunction. J. Heart Lung Transplant..

[27] Perna A.F., Satta E., Acanfora F., Lombardi C., Ingrosso D., de Santo N.G. (2006). Increased plasma protein homocysteinylation in hemodialysis patients. Kidney Int..

[28] Perna A.F., Acanfora F., Luciano M.G., Pulzella P., Capasso R., Satta E., Cinzia L., Pollastro R.M., Iannelli S., Ingrosso D. (2007). Plasma protein homocysteinylation in uremia. Clin. Chem. Lab. Med..

[29] Jakubowski H. (2004). Molecular basis of homocysteine toxicity in humans. Cell. Mol. Life. Sci..

[30] Sass J.O., Nakanishi T., Sato T., Sperl W., Shimizu A. (2003). S-homocysteinylation of transthyretin is detected in plasma and serum of humans with different types of hyperhomocysteinemia. Biochem. Biophys. Res. Commun..

[31] Lim A., Sengupta S., McComb M.E., Theberge R., Wilson W.G., Costello C.E., Jacobsen D.W. (2003). In vitro and in vivo interactions of homocysteine with human plasma transthyretin. J. Biol. Chem..

[32] Jakubowski H. (1991). Proofreading in vivo: Editing of homocysteine by methionyl-tRNA synthetase in the yeast Saccharomyces cerevisiae. EMBO J..

[33] Jakubowski H., Goldman E. (1993). Synthesis of homocysteine thiolactone by methionyl-tRNA synthetase in cultured mammalian cells. FEBS Lett..

[34] Jakubowski H. (2016). Aminoacyl-tRNA synthetases and the evolution of coded peptide synthesis: The Thioester World. FEBS Lett..

[35] Perla-Kajan J., Jakubowski H. (2010). Paraoxonase 1 protects against protein *N*-homocysteinylation in humans. FASEB J..

[36] Perla-Kajan J., Stanger O., Luczak M., Ziolkowska A., Malendowicz L.K., Twardowski T., Lhotak S., Austin R.C., Jakubowski H. (2008). Immunohistochemical detection of *N*-homocysteinylated proteins in humans and mice. Biomed. Pharmacother..

[37] Jakubowski H. (2008). New method for the determination of protein N-linked homocysteine. Anal. Biochem..

[38] Uji Y., Motomiya Y., Hanyu N., Ukaji F., Okabe H. (2002). Protein-bound homocystamide measured in human plasma by HPLC. Clin. Chem..

[39] Jakubowski H. (2002). Homocysteine is a protein amino acid in humans. Implications for homocysteine-linked disease. J. Biol. Chem..

[40] Jakubowski H., Perla-Kajan J., Finnell R.H., Cabrera R.M., Wang H., Gupta S., Kruger W.D., Kraus J.P., Shih D.M. (2009). Genetic or nutritional disorders in homocysteine or folate metabolism increase protein *N*-homocysteinylation in mice. FASEB J..

[41] Jakubowski H. (2001). Protein *N*-homocysteinylation: Implications for atherosclerosis. Biomed. Pharmacother..

[42] Paoli P., Sbrana F., Tiribilli B., Caselli A., Pantera B., Cirri P., de Donatis A., Formigli L., Nosi D., Manao G. (2010). Protein *N*-homocysteinylation induces the formation of toxic amyloid-like protofibrils. J. Mol. Biol..

[43] Undas A., Perla J., Lacinski M., Trzeciak W., Kazmierski R., Jakubowski H. (2004). Autoantibodies against *N*-homocysteinylated proteins in humans: Implications for atherosclerosis. Stroke J. Cereb. Circ..

[44] Sauls D.L., Lockhart E., Warren M.E., Lenkowski A., Wilhelm S.E., Hoffman M. (2006). Modification of fibrinogen by homocysteine thiolactone increases resistance to fibrinolysis: A potential mechanism of the thrombotic tendency in hyperhomocysteinemia. Biochemistry.

[45] Sauls D.L., Warren M., Hoffman M. (2011). Homocysteinylated fibrinogen forms disulfide-linked complexes with albumin. Thromb. Res..

[46] Liu M., Zhang Z., Zang T., Spahr C., Cheetham J., Ren D., Zhou Z.S. (2013). Discovery of Undefined Protein Crosslinking Chemistry: A Comprehensive Methodology Utilizing ^18^O-labeling and Mass Spectrometry. Anal. Chem..

[47] Chen T.S., Nayak N., Majee S.M., Lowenson J., Schafermeyer K.R., Eliopoulos A.C., Lloyd T.D., Dinkins R., Perry S.E., Forsthoefel N.R. (2010). Substrates of the Arabidopsis thaliana Protein Isoaspartyl Methyltransferase 1 Identified Using Phage Display and Biopanning. J. Biol. Chem..

[48] Ni W., Dai S., Karger B.L., Zhou Z.S. (2010). Analysis of isoaspartic Acid by selective proteolysis with Asp-N and electron transfer dissociation mass spectrometry. Anal. Chem..

[49] Liu M., Cheetham J., Cauchon N., Ostovic J., Ni W., Ren D., Zhou Z.S. (2012). Protein isoaspartate methyltransferase-mediated ^18^O-labeling of isoaspartic acid for mass spectrometry analysis. Anal. Chem..

[50] Dai S., Ni W., Patananan A.N., Clarke S.G., Karger B.L., Zhou Z.S. (2013). Integrated proteomic analysis of major isoaspartyl-containing proteins in the urine of wild type and protein l-isoaspartate *O*-methyltransferase-deficient mice. Anal. Chem..

[51] Liu M., Zhang Z., Cheetham J., Ren D., Zhou Z.S. (2014). Discovery and characterization of a photo-oxidative histidine-histidine cross-link in IgG1 antibody utilizing ^18^O-labeling and mass spectrometry. Anal. Chem..

[52] Liu S., Moulton K.R., Auclair J.R., Zhou Z.S. (2016). Mildly acidic conditions eliminate deamidation artifact during proteolysis: Digestion with endoprotease Glu-C at pH 4.5. Amino Acids.

[53] Zhou Z.S., Smith A.E., Matthews R.G. (2000). l-selenohomocysteine: One-step synthesis from l-selenomethionine and kinetic analysis as substrate for methionine synthases. Bioorg. Med. Chem. Lett..

[54] Zang T., Lee B.W., Cannon L.M., Ritter K.A., Dai S., Ren D., Wood T.K., Zhou Z.S. (2009). A naturally occurring brominated furanone covalently modifies and inactivates LuxS. Bioorg. Med. Chem. Lett..

[55] Wang Z., Rejtar T., Zhou Z.S., Karger B.L. (2010). Desulfurization of cysteine-containing peptides resulting from sample preparation for protein characterization by mass spectrometry. Rapid Commun. Mass Spectrom..

[56] Zhao G., Zhou Z.S. (2001). Vinyl sulfonium as novel proteolytic enzyme inhibitor. Bioorg. Med. Chem. Lett..

[57] Chumsae C., Gifford K., Lian W., Liu H., Radziejewski C.H., Zhou Z.S. (2013). Arginine modifications by methylglyoxal: Discovery in a recombinant monoclonal antibody and contribution to acidic species. Anal. Chem..

[58] Sibrian-Vazquez M., Escobedo J.O., Lim S., Samoei G.K., Strongin R.M. (2010). Homocystamides promote free-radical and oxidative damage to proteins. Proc. Natl. Acad. Sci. USA.

[59] Sharma G.S., Kumar T., Singh L.R. (2014). *N*-homocysteinylation induces different structural and functional consequences on acidic and basic proteins. PLoS ONE.

[60] Gurda D., Handschuh L., Kotkowiak W., Jakubowski H. (2015). Homocysteine thiolactone and *N*-homocysteinylated protein induce pro-atherogenic changes in gene expression in human vascular endothelial cells. Amino Acids.

[61] Jakubowski H. (2006). Pathophysiological consequences of homocysteine excess. J. Nutr..

[62] Jakubowski H. (2016). Quantification of urinary *S*- and *N*-homocysteinylated protein and homocysteine-thiolactone in mice. Anal. Biochem..

[63] Ferguson E., Parthasarathy S., Joseph J., Kalyanaraman B. (1998). Generation and initial characterization of a novel polyclonal antibody directed against homocysteine thiolactone-modified low density lipoprotein. J. Lipid Res..

[64] Zang T., Dai S., Chen D., Lee B.W., Liu S., Karger B.L., Zhou Z.S. (2009). Chemical methods for the detection of protein *N*-homocysteinylation via selective reactions with aldehydes. Anal. Chem..

[65] Fathe K., Person M.D., Finnell R.H. (2015). The application of a chemical determination of *N*-homocysteinylation levels in developing mouse embryos: Implication for folate responsive birth defects. J. Nutr. Biochem..

[66] Hodgson N.W., Waly M.I., Al-Farsi Y.M., Al-Sharbati M.M., Al-Farsi O., Ali A., Ouhtit A., Zang T.Z., Zhou Z.S., Deth R.C. (2014). Decreased glutathione and elevated hair mercury levels are associated with nutritional deficiency-based autism in Oman. Exp. Biol. Med..

[67] Alfaro J.F., Gillies L.A., Sun H.G., Dai S., Zang T., Klaene J.J., Kim B.J., Lowenson J.D., Clarke S.G., Karger B.L. (2008). Chemo-enzymatic detection of protein isoaspartate using protein isoaspartate methyltransferase and hydrazine trapping. Anal. Chem..

[68] Guttormsen A.B., Schneede J., Ueland P.M., Refsum H. (1996). Kinetics of total plasma homocysteine in subjects with hyperhomocysteinemia due to folate or cobalamin deficiency. Am. J. Clin. Nutr..

[69] Herrmann M., Wildemann B., Wagner A., Wolny M., Schorr H., Taban-Shomal O., Umanskaya N., Ross S., Garcia P., Hübner U. (2009). Experimental folate and vitamin B_12_ deficiency does not alter bone quality in rats. J. Bone Miner. Res..

[70] Stangl G.I., Schwarz F.J., Jahn B., Kirchgessner M. (2000). Cobalt-deficiency-induced hyperhomocysteinaemia and oxidative status of cattle. Br. J. Nutr..

[71] Troen A.M., Shea-Budgell M., Shukitt-Hale B., Smith D.E., Selhub J., Rosenberg I.H. (2008). B-vitamin deficiency causes hyperhomocysteinemia and vascular cognitive impairment in mice. Proc. Natl. Acad. Sci. USA.

[72] Eberhardt R.T., Forgione M.A., Cap A., Leopold J.A., Rudd M.A., Trolliet M., Heydrick S., Stark R., Klings E.S., Moldovan N.I. (2000). Endothelial dysfunction in a murine model of mild hyperhomocyst(e)inemia. J. Clin. Investig..

[73] Weiss N., Heydrick S., Zhang Y.Y., Bierl C., Cap A., Loscalzo J. (2002). Cellular redox state and endothelial dysfunction in mildly hyperhomocysteinemic cystathionine beta-synthase-deficient mice. Arterioscler. Thromb. Vasc. Biol..

[74] Wiese S., Reidegeld K.A., Meyer H.E., Warscheid B. (2007). Protein labeling by iTRAQ: A new tool for quantitative mass spectrometry in proteome research. Proteomics.

[75] Camps J., Marsillach J., Joven J. (2009). The paraoxonases: Role in human diseases and methodological difficulties in measurement. Crit. Rev. Clin. Lab. Sci..

[76] Miller L.L., Bly C.G., Watson M.L., Bale W.F. (1951). The dominant role of the liver in plasma protein synthesis; a direct study of the isolated perfused rat liver with the aid of lysine-epsilon-C14. J. Exp. Med..

[77] Sikora M., Marczak L., Kubalska J., Graban A., Jakubowski H. (2014). Identification of *N*-homocysteinylation sites in plasma proteins. Amino Acids.

[78] Araki A., Sako Y. (1987). Determination of free and total homocysteine in human plasma by high-performance liquid chromatography with fluorescence detection. J. Chromatogr. B.

