# Supplementary material for: Comparison of Protein N-Homocysteinylation in Rat Plasma under Elevated Homocysteine Using a Specific Chemical Labeling Method
Source: Molecules. 2016 Sep 8;21(9):1195. doi: 10.3390/molecules21091195 (PMC5292613; doi:10.3390/molecules21091195)
Supplement: Supplementary file 1 [file molecules-21-01195-s001.pdf]

Supplementary Materials: Comparison of Protein *N*-Homocysteinylation in Rat Plasma under Elevated Homocysteine Using a Specific Chemical Labeling Method

Tianzhu Zang, Ligi Paul Pottenplackel, Diane E. Handy, Joseph Loscalzo, Shujia Dai, Richard C. Deth, Zhaohui Sunny Zhou and Jisheng Ma

1. Materials and Methods

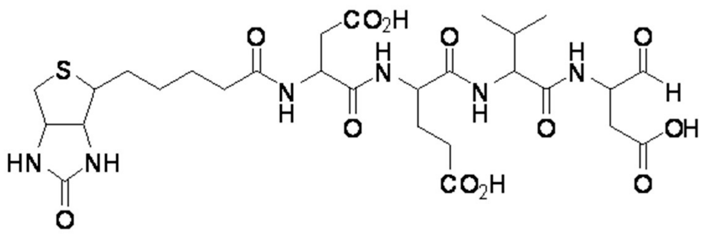

Figure S1. Structure of biotin aldehyde tag (CAS registry number: 178603-73-1).

2. Optimized Conditions for Biotin-Aldehyde Labeling

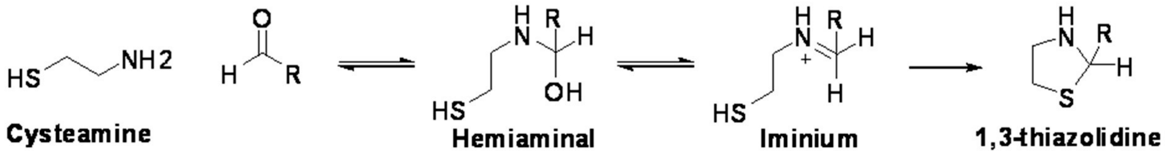

Figure S2. Formation of 1,3-thiazolidine between cysteamine and aldehyde.

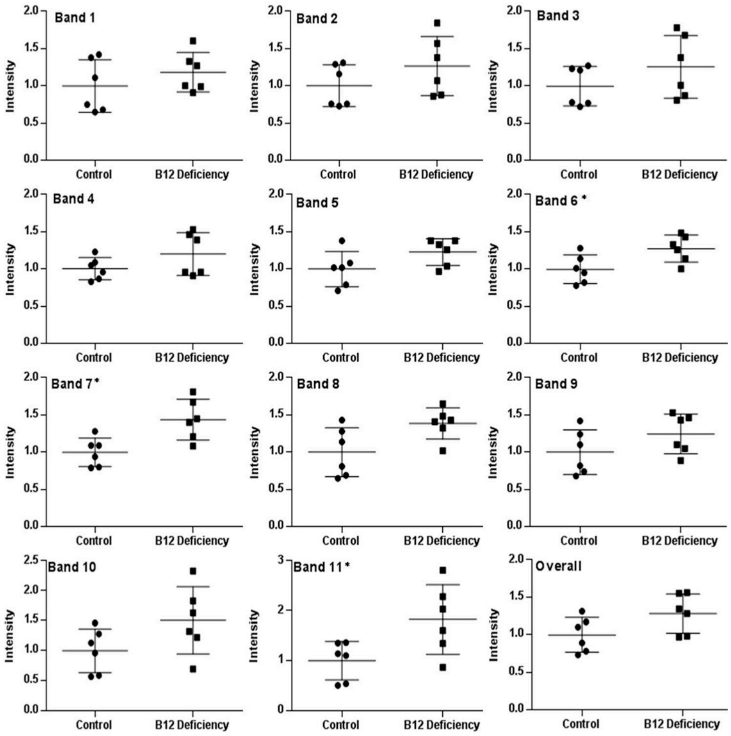

Figure S3. Intensities of protein *N*-homocyteinylation from rat plasma by *t*-test assay. Overall intensity is for all proteins in the complete lanes. *: protein band with a statistically significant change ($p < 0.05$).

4. Detection of Protein *N*-Homocysteinylation in Plasma from Mice with Heterozygous CBS-Deficiency (*Cbs*$^{+/-}$)

4.1. Mice Plasma

Plasma collected from mice with wild-type CBS (*Cbs*$^{+/+}$) and heterozygous CBS-deficiency (*Cbs*$^{+/-}$) (two in each group) were from Drs. Diane Handy and Joseph Loscalzo at Harvard Medical School and the protocol was approved by the Harvard Medical Area Standing Committee on Animals. Heterozygous CBS-deficient mice have mildly elevated plasma "total Hcy" (5.8 ± 0.7 μM) compared to their wild type littermates (3.6 ± 0.4 μM), an increase of 1.6-fold [1,2]. Mice plasma (4 mg/mL protein, final concentration) was used for the assay. The experimental procedure was described in "Materials and Methods" in the main text.

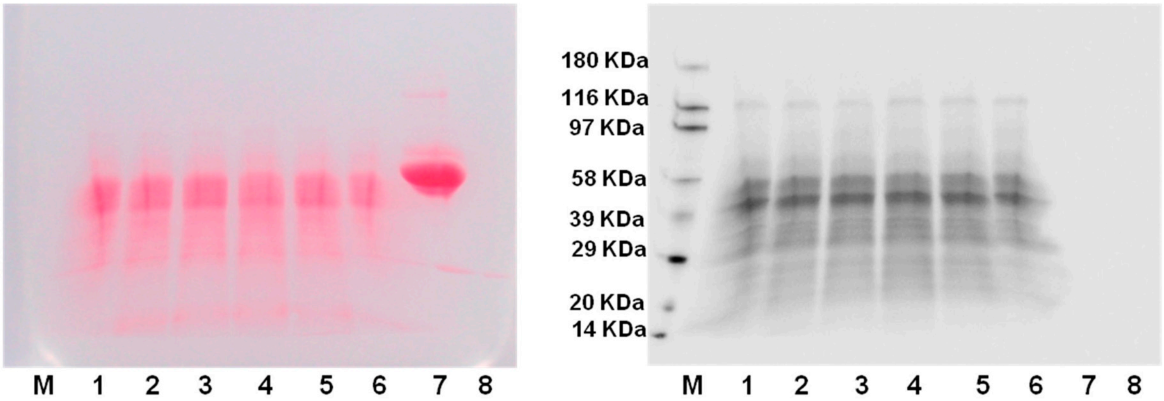

M	1	2	3	4	5	6	7
Marker	WT	Het	Het	WT	Het	Het	Het w/o Biotin

WT: wild type; Het: heterozygous CBS deficient type.

Figure S4. Western-blotting image (**right**) and Ponceau-S staining image (**left**) of mice plasma.

References

1. Eberhardt, R.T.; Forgione, M.A.; Cap, A.; Leopold, J.A.; Rudd, M.A.; Trolliet, M.; Heydrick, S.; Stark, R.; Klings, E.S.; Moldovan, N.I.; et al. Endothelial dysfunction in a murine model of mild hyperhomocyst(e)inemia. *J. Clin. Investig.* **2000**, *106*, 483–491.
2. Weiss, N.; Heydrick, S.; Zhang, Y.Y.; Bierl, C.; Cap, A.; Loscalzo, J. Cellular redox state and endothelial dysfunction in mildly hyperhomocysteinemic cystathionine beta-synthase-deficient mice. *Arterioscler. Thromb. Vasc. Biol.* **2002**, *22*, 34–41.
